# An *in vivo* Like Micro-Carcinoma Model

**DOI:** 10.3389/fonc.2019.00410

**Published:** 2019-05-22

**Authors:** Sandra Camargo, Yulia Shamis, Assaf Assis, Eduardo Mitrani

**Affiliations:** ^1^Department of Cell and Developmental Biology, The Hebrew University of Jerusalem, Jerusalem, Israel; ^2^Department of Developmental and Regenerative Biology, Department of Oncological Sciences, Icahn School of Medicine at Mount Sinai, New York, NY, United States

**Keywords:** carcinoma, tumor microenvironment, tumor micro-culture, tumor model, decellularized scaffolds

## Abstract

We here present a novel micro-system which allows to reconstitute an in vivo lung carcinoma where the various constituting epithelial and/or stromal structural and/or cellular components can be incorporated at will. In contrast to various “organs on a chip” the model is based on the observation that in nature, epithelial cells are always supported by a connective tissue or stroma. The model is based on acellular micro-scaffolds of microscopic dimensions which enable seeded cells to obtain gases and nutrients through diffusion thus avoiding the need for vascularization. As a proof of concept, we show that in this model, Calu-3 cells can form a well-organized, continuous, polarized, one-layer epithelium lining the stromal derived alveolar cavities, and express a different pattern of tumor-related genes than when grown as standard monolayer cultures on plastic culture dishes. To our knowledge, this model, introduces for the first time a system where the function of carcinogenic cells can be tested in vitro in an environment that closely mimics the natural in vivo situation.

## Introduction

The intention of the model proposed here is to recreate as far as possible in an *in vitro* system, the *in vivo* conditions where normal epithelial stromal interactions are preserved. Epithelial-stromal interactions are necessary for homeostasis in any epithelial-containing organ (1). The tissue microarchitecture plays a main role in cell differentiation, organization, function, and molecular signaling, not only in normal conditions but also in diseases such as cancer (1). During development, the interaction between parenchymal cells and their stroma is fundamental in determining the future of the epithelial cells (2). In adult tissues two-way communication between cells and their surroundings is well established (3). Some researchers have suggested that in tumors this cross-talk is disrupted and stroma could have a key role in carcinogenesis (4).

Three dimensional *in vitro* tumor models allow to study not only cell proliferation, organization, and differentiation but also the molecular pathways important for a better understanding of epithelium-stroma interactions during carcinogenesis (5). The emerging evidence suggests that the local tumor microenvironment could regulate positively or negatively the properties of malignant epithelial cells. Some studies have displayed how the phenotype of tumorigenic cells could be reverted by correcting the extracellular matrix-receptor signaling (6). Others, have shown how the loss of appropriate molecular factors whose signaling must be integrated in order to effect an organized and functional tissue morphology, could influence the formation of aberrant cell phenotypes (7, 8). It has recently been shown that reestablishing of tissue organization by cell surface receptors such as the Integrins that mediate cell–cell and cell–extracellular matrix interactions could override some malignant features (9). Other authors have focused on explore epithelial-stromal cells interactions during carcinogenesis, by showing that growth factors and matrix enzymes secreted by fibroblasts in the stroma could influence malignant conditions (10–12). Besides, some changes in physical features such as extra cellular matrix architecture and stiffening due to abnormal matrix remodeling may also be involved in tumor development (10, 13–15).

We strongly believe that in contrast to other simplified approaches that claim to obtain an “organ on a chip,” in order to study *in vitro* the tumor in context it is necessary to mimic the environmental conditions that reflect the natural situation. Indeed, some researchers have demonstrated the importance of 3D culture systems to study the interplay between the tumor epithelium and its stroma (1). Specifically in cancer, this kind of system has allowed investigating important issues as development, progression, metastasis, cell resistance to apoptosis and to test different therapeutic approaches (13, 15–17). Other investigators, using simple systems composed of a basement membrane extract as a 3D support for endometrial cancer cells have also shown how even this type extra cellular matrix can inhibit expression of TGF-β by the endometrial cells (18). Indeed, there is a considerable effort to recreate the ECM structure and its protein composition (5). To that extent several kinds of synthetic biomaterial matrices as polymers, and natural matrices as decellularized organs, porcine intestinal submucosa, collagen, or cell-derived products, have been used as scaffolds for 3D cultures (5, 16, 19–25). In most of these cultures it is necessary to use a bioreactor that allows the oxygenation for the adequate cells growth (25).

Since, epithelial cells are always supported by a connective tissue or stroma, our group has been developing three-dimensional *in vitro* systems based on decellularized scaffolds of microscopic dimensions. Such micro-scaffolds have been shown to serve as bases for reengineering complex organ-like structures *in vitro* which contain, in addition to the parenchyma most of the extracellular components found in the original stroma (26–28). In fact in previous works we have started to characterize by MS/MS different types of stroma and found that for instance that lung, pancreas or kidney, each contain at least 55 different ECM components (26, 29).

An important feature of the micro-scaffolds which constitute the basis for our model system is that they are never more than 300 microns in thickness. These dimensions ensure that any seeded cell can obtain gases and nutrients by simple diffusion and thus eliminating the need for vascularization. Using such approach it has been shown for example that expanded alveolar cells when seeded into an acellular, lung-derived micro-scaffold can re-populate the matrix and generate proper alveolar structures which are structurally and biochemically indistinguishable from the original tissue (26). Similarly whole or dissociated islets of Langerhans when seeded into appropriate micro-scaffolds secrete high levels of insulin in a regulated manner for several months *in vitro* and can rescue hyperglycemic animals using a relatively small number of cells (27, 28).

Based on those findings and on the observation that through evolution each epithelial-containing organ has a specific stroma, we now propose a new model which allows us to study the interactions between carcinogenic epithelial cells as they interact with a highly complex organ-specific stroma containing various types of cells and several hundred different ECM components organized in a functional structure.

## Materials and Methods

Animal experiments were performed under the guidelines and approval of the Animal Care and Use Committee, The Faculty of Science of the Hebrew University, Jerusalem, Israel (Permit number NS-17-15219-3).

### Cells

#### Calu-3 Cells

Calu-3 cells (ATCC, HTB-55), are an epithelial immortalized line derived from a human lung adenocarcinoma. Calu-3 cells were maintained in Petri tissue culture dishes (100 mm) in culture medium containing Dulbecco's Modified Eagle's medium, DMEM, supplemented with 10% of fetal calf serum, 1% of penicillin (100 U/ml)-streptomycin (1 mg/mL), and 1% of L-glutamine (2 mM) at 37°C in 5% CO_2_. All reagents are from Biological Industries, Israel. The medium was changed each 2–3 days. Passage numbers 19–22 were used for the experiments. Any test for Mycoplasma was done.

#### Isolation of Primary Fibroblasts

A piece of skin of 1 cm^2^ was taken from a rat strain Sabra. The skin was maintained in Dulbecco's Phosphate Buffered Saline, PBS (Biological Industries, Israel), while it was divided and cut with a scalpel in 10 pieces. Skin fragments were washed once for 30 min at 4°C with 3 ml of PBS supplemented with 1% of penicillin-streptomycin, three times for 10 min with 3 ml of PBS without antibiotic and once with culture medium as was described before. These skin pieces were cultured in a 12-well plate (2–3 pieces/well). The dermal side of each skin fragment was seeded in touch with the well surface to allow adhesion for 30 s without medium. Finally, 1 mL of culture medium was added carefully to avoid detaching the skin from the well bottom and incubated at 37°C in 5% CO_2_. The medium was replaced every 2–3 days. After 1 week, when the wells had become 80–90% confluent, cells were detached with trypsin solution and plated in a tissue culture dish (100 mm) or used for subsequent experiments. Passage numbers 2–4 were used for the experiments.

### Preparation of Lung-Derived Acellular Micro-Scaffolds

A method for preparing micro-scaffolds has been previously described (24–26).

Briefly, lungs were taken from a rat strain Sprague Dawley, and maintained in PBS while they were cut. Then, each lobe was cut transversely into 300-μm-thick fragments, using a Sorvall TC-2 tissue chopper and put again in PBS as described previously (26). After cutting the fragments were inserted into a 250 mL sterile filter (pore size, 0.22 μm) under continuous shaking, organ fragments and washed three times with 1M NaCl (Bio-lab, Israel) for 20 min each. This was followed by four short 3 min washes with distilled water and two incubations with 0.5% Triton-X100 (Sigma, Israel) for 15 min each. This was followed by six washes with distilled water for 20 min each and then stored with PBS supplemented with 1% of penicillin (100 U/mL)-streptomycin (1 mg/mL) and 1 mL Gentamicin (50 mg/mL) (Sigma, Israel) for 20 min. For a period of <2 weeks until used (27).

### Seeding of Cells Onto Lung-Derived Micro-Scaffolds

**Figure 1:**
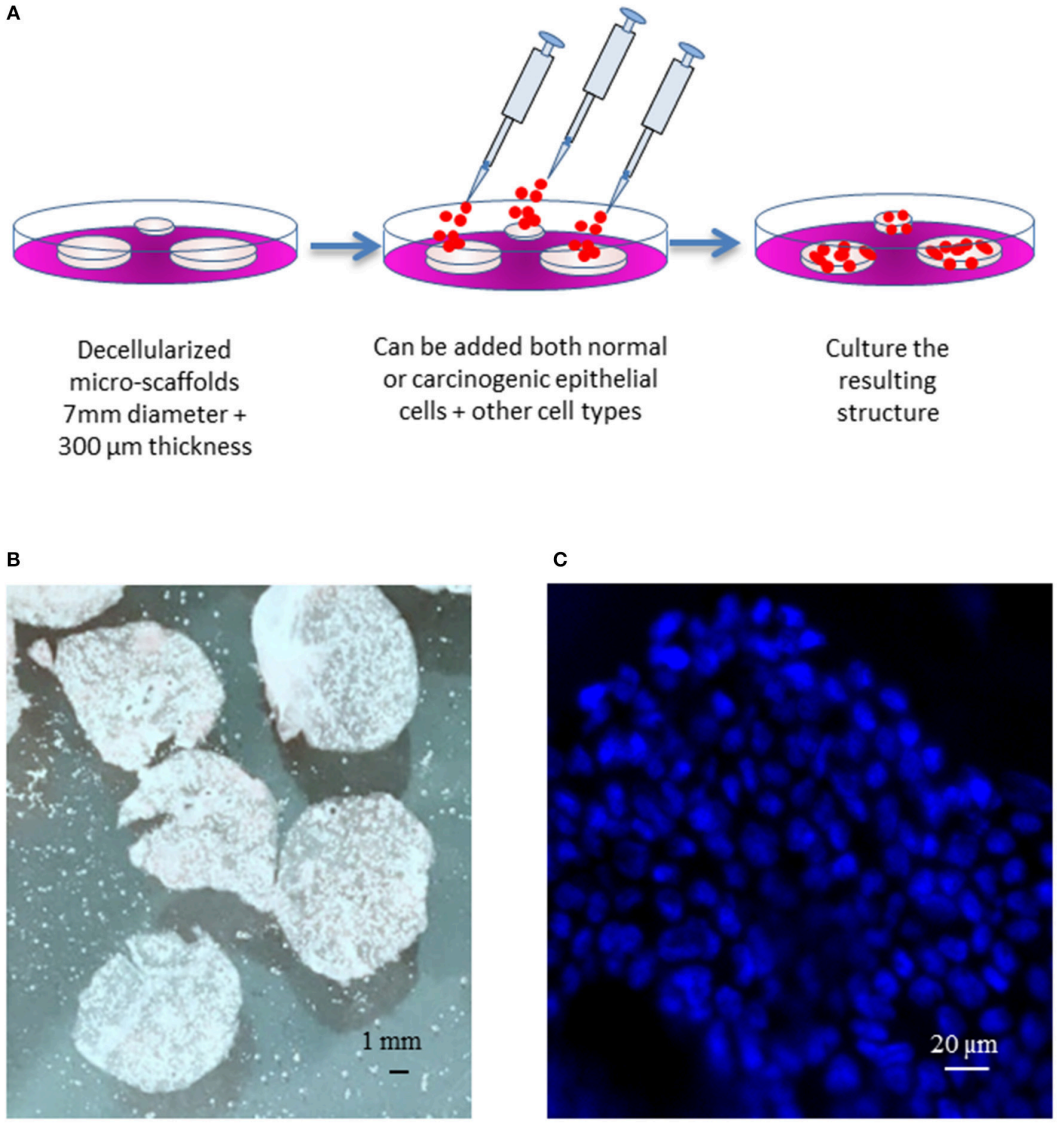
General model. **(A)** Schematic diagram of the three-dimensional culture system. **(B)** Decellularized micro-scaffolds. **(C)** DAPI staining of micro-scaffolds seeded with Calu-3 cells. *Scale bars* **(B)** 1 mm, **(C)** 20 μm.

Micro-scaffolds were first washed three times in PBS and inserted into wells on 12 wells plate containing 750 uL of culture medium (5 micro-scaffolds/well). Calu-3 cells were seeded on each micro-scaffold by carefully layering 50 uL of culture media containing 4 × 10^4^ Calu-3 on top of each micro-scaffold. The seeded micro-scaffolds were incubated at 37°C, 5% CO_2_. After 3 days, primary fibroblasts were seeded at a cell concentration of 8 × 10^3^ cells/micro-scaffold over some constructs of micro-scaffolds with Calu-3. The medium was changed every 2–3 days. Controls of cells were seeded on plastic culture dishes without micro-scaffolds using the same culture medium and maintained at the same conditions. The general method of seeding on the micro-scaffolds is explained in Figure 1.

### Viability Assay

To test viability of cells on micro-scaffolds, the constructs were washed in PBS and incubated for 10–20 min at 37°C, 5% CO_2_ in 0.5 mg\ mL MTT (3- (4,5-Dimethyl-2-thiazolyl)-2,5-diphenyl-2H-tetrazolium bromide), (Sigma, Israel).

### DAPI Staining

Constructs were fixed 4% paraformaldehyde, then nuclei were stained with 5 μg/ml of 4-6diamino-2-phenylindole dihydrochloride (DAPI, Sigma) in PBS.

### H&E Staining

Constructs were fixed 4% paraformaldehyde, then embedded in paraffin and 8 μm sections prepared and stained with hematoxylin/eosin (H&E) according to routine histological methods.

### Electron Microscopy Analysis

Constructs were fixed in 1% formaldehyde and 2.5% glutaraldehyde, postfixed in 1% osmium tetroxide, dehydrated, and embedded in Epon using standard techniques. Ultrathin sections (60–80 nm thick) were prepared using an Ultratome III (LKB). The sections were collected on 200-mesh thin bar grids, counterstained with uranyl acetate and lead citrate, and examined using a Philips CM 120 transmission electron microscope (26).

### Molecular Analysis

For RNA extraction, seeded micro-scaffolds were homogenized using a motor-driven pellet pestle (Sigma, Israel) and follow the Tri-Reagent protocol (Sigma, Israel). Then, cDNA preparation was done according to the Reverse Transcription Kit (Applied Biosystems, Israel). Afterwards, qRT-PCR was done using the Fast SYBR^TM^ Green Master Mix Kit protocol (Applied Biosystems, Israel) as follows, 10 μL of 2X SYBR® Green PCR Master Mix, 2 μL of primer mix (10 μM of Forward primer and 10 μM of Reverse primer), 6 μL of ultrapure water (Sigma, Israel) and 2 μL of cDNA to reach a final volume of 20 μL per well, in a 96 well multiplate. Primers sets for qRT-PCR are summarized in Table 1. It should be noted that primers chosen will only amplify human but not rar derived cDNAs.

**Table 1:** Primers sets for qRT-PCR.

**Primer name**	**Forward (5^**′**^-3^**′**^)**	**Reverse (5^**′**^-3^**′**^)**
**HOUSEKEEPING GENE**		
*TBP*	GCCAAGAGTCAAGAACAG	GAAGTCCAAGAACTTAGCTG
**ONCOGENES**		
*AKT1*	TTCTCCAGCTTGAGGTC	AAGTACTCTTTCCAGACCC
*BCL-XL*	ATCTCTTTCTCTCCCTTCAG	CTTTCTGGGAAAGCTTGTAG
*MET*	CATGTGAATTTTCTCCTGGAC	ATCTTCTTCCCAGTGATAACC
*MYC*	TGAGGAGGAACAAGAAGATG	ATCCAGACTCTGACCTTTTG
*EGFR*	AGTGCCTGAATACATAAACC	GTAGTGTGGGTCTCTGC
*EPCAM*	GTATGAGAAGGCTGAGATAAAG	CTTCAAAGATGTCTTCGTCC
*ERBB2*	CCAGCCTGAATATGTGAAC	CCCCAAAGGCAAAAACG
*STAT3*	GGTACATCATGGGCTTTATC	TTTGCTGCTTTCACTGAATC
*BRAF*	ATATCTGGAGGCCTATGAAG	CTGAAAGAGATGAAGGTAGC
*BIRC5*	CATCTCTACATTCAAGAACTGG	CCTTGAAGCAGAAGAAACAC
**TUMOR SUPPRESSORS**		
*FAS/CD95*	CTGTCCTCCAGGTGAAAG	TGTACTCCTTCCCTTCTTG
*PTEN*	GGCTAAGTGAAGATGACAATC	GTTACTCCCTTTTTGTCTCTG
*FHIT*	GAAATCCACTGAGAACAGTC	ACCTTCTTTCTCTTTCTCTCC
*TP53*	ACCTATGGAAACTACTTCCTG	ACCATTGTTCAATATCGTCC
*P14^*ARF*^*	CCCTCGTGCTGATGCTACTG	CATCATGACCTGGTCTTCTAGGAA

Expression levels were calculated by the ΔC_t_ method after normalizing the genes with TATA—box binding protein (TBP) as follows: for experimental samples (Calu-3 cells seeded on micro-scaffolds) and for the experimental control (Calu-3 seeded on plastic culture dishes), the average of each target gene CT was normalized with the average of the housekeeping gene CT, for each time point. At this point there are two values, the delta CT value of the target gene for experimental samples (ΔCTS), and the delta CT value of the target gene for the experimental control (ΔCTC). Then, the difference between ΔCTS and ΔCTC was calculated, which is the Double Delta CT Value (ΔΔCT). Finally, the value of 2^∧^−ΔΔCT was calculated to get the expression fold change.

### Statistical Analyses

All analyses were performed in at least duplicate. Statistical analysis was performed using *t*-test. A *p* ≤ 0.05 was considered significant and *p* ≤ 0.01 highly significant.

## Results

### Reestablishing of Tissue Organization

Calu-3 cells were seeded alone or in co-culture with rat primary fibroblast on natural micro-scaffolds derived from rat-lung. In parallel, as controls Calu-3 cells and their co-culture with fibroblasts were also seeded on culture dishes under the same culture conditions. Samples from the resulting constructs and their respective controls were taken at different time points. The three-dimensional organization and morphology of Calu-3 cells seeded on micro-scaffolds were examined by histological analysis.

**Figure 2:**
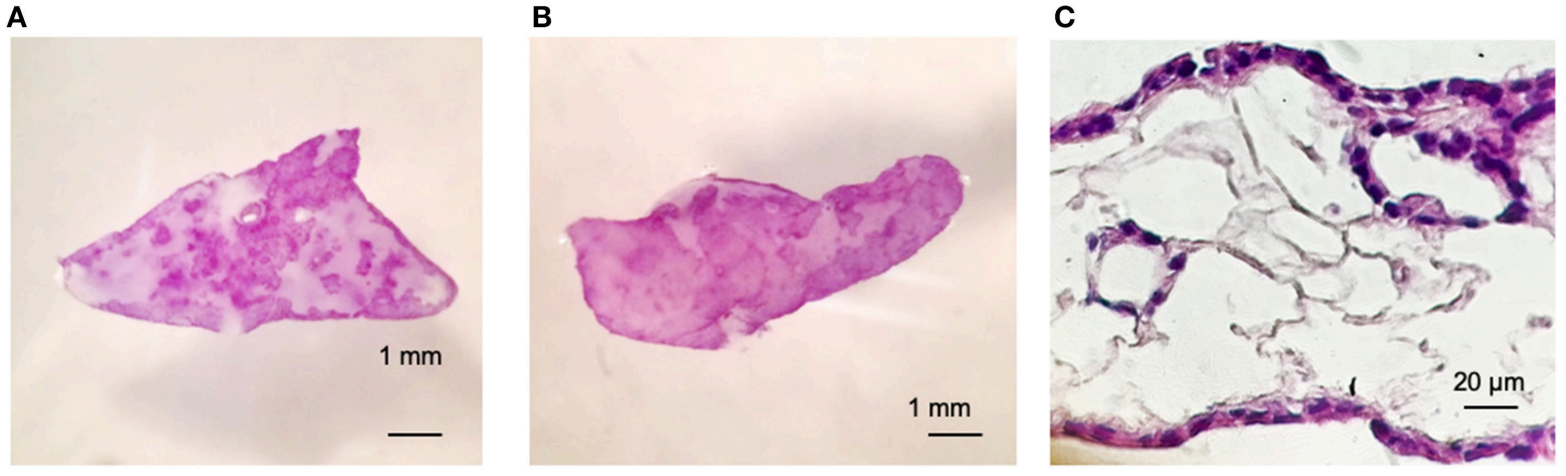
Calu-3 cells can organize themselves into a continuous one-layer epithelium lining the major micro-scaffold cavities. Cell viability by MTT assay at day 15 of **(A)** Calu-3 cells on micro-scaffolds and **(B)** Calu-3 cells and fibroblast on micro-scaffolds. Histological sections at day 15 of **(C)** Calu-3 cells seeded on micro-scaffolds. *Scale bars* **(A,B)** 1 mm, **(C)** 20 μm.

Figures 2A,B shows viability of Calu-3 cells by MTT (3- (4,5-Dimethyl-2-thiazolyl)-2,5-diphenyl-2H-tetrazolium bromide) staining and in both cases, alone or in co-culture with fibroblasts. Cells populated the matrix and remained viable for at least 30 days –the longest period tested-. As the culture progresses, the constructs containing fibroblasts contracted while those with only the Calu-3 cells remained flat. Histological sections showed Calu-3 cells can penetrate into the deepest areas of the scaffold and not just cover the outer surface (Figure 2C). We have previously shown that lung derived alveolar cells can populate the lung micro-scaffolds and arrange themselves into highly organized epithelia recreating alveolar structures (26). Surprisingly, as shown in Figure 2C, Calu-3 tumorigenic cell line was found to organize itself into a continuous one-layer epithelium lining the major cavities. When lung micro-scaffolds were seeded with normal fibroblasts and Calu-3 cells, a similar pattern of penetration and occupancy was observed.

**Figure 3:**
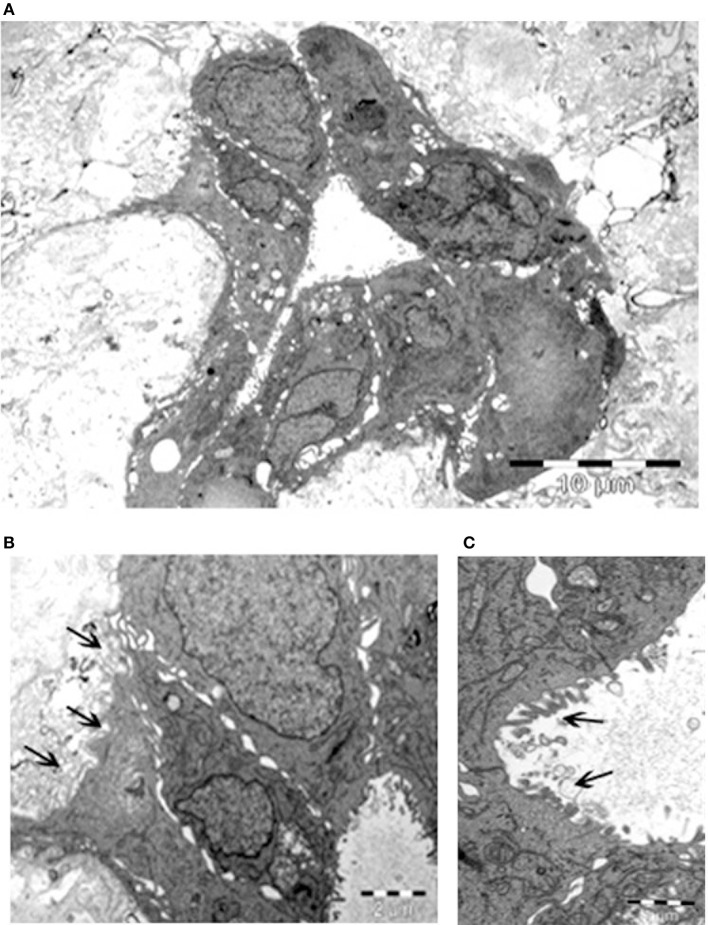
Calu-3 cells form a polarized single layer epithelium. Transmission electron microscopy of **(A)** epithelium formed by the Calu-3 cells, expanded around the alveolar cavity of the matrix. **(B)** Higher magnification of **(A)** demonstrates basal surface of the cells lined by a basement membrane (arrows) and cell contacts. **(C)** Higher magnification of **(B)** shows the apical surface of the cells covered by microvilli (arrows). *Scale bars* 10 μm **(A)**, 2 μm **(B)**, and 1 μm **(C)**.

The remarkable, unexpected organization of Calu-3 cells seeded onto lung micro-scaffolds was also examined by electron microscopy (Figure 3). Calu-3 cells were found to form a polarized single layer epithelium that expanded around the alveolar cavities of the matrix. Furthermore, the apical surface of the cells was found to be covered by microvilli (marked by arrows on Figure 3C) while the basal surface of the cells was found to be lined by a basement membrane (marked by arrows on Figure 3B).

### Adenocarcinoma Cells Display a Less Malignant Phenotype When Seeded Into Natural Micro-Scaffolds

As an initial screen, expression of several key genes reported to be either upregulated or downregulated during carcinogenesis were examined by qRT-PCR. Expression was tested in Calu-3 cells seeded on micro-scaffolds and normalized against Calu-3 cells seeded on standard culture dishes. Analysis was done at 4 different time points, day 3, 7, 15, and 30.

**Figure 4:**
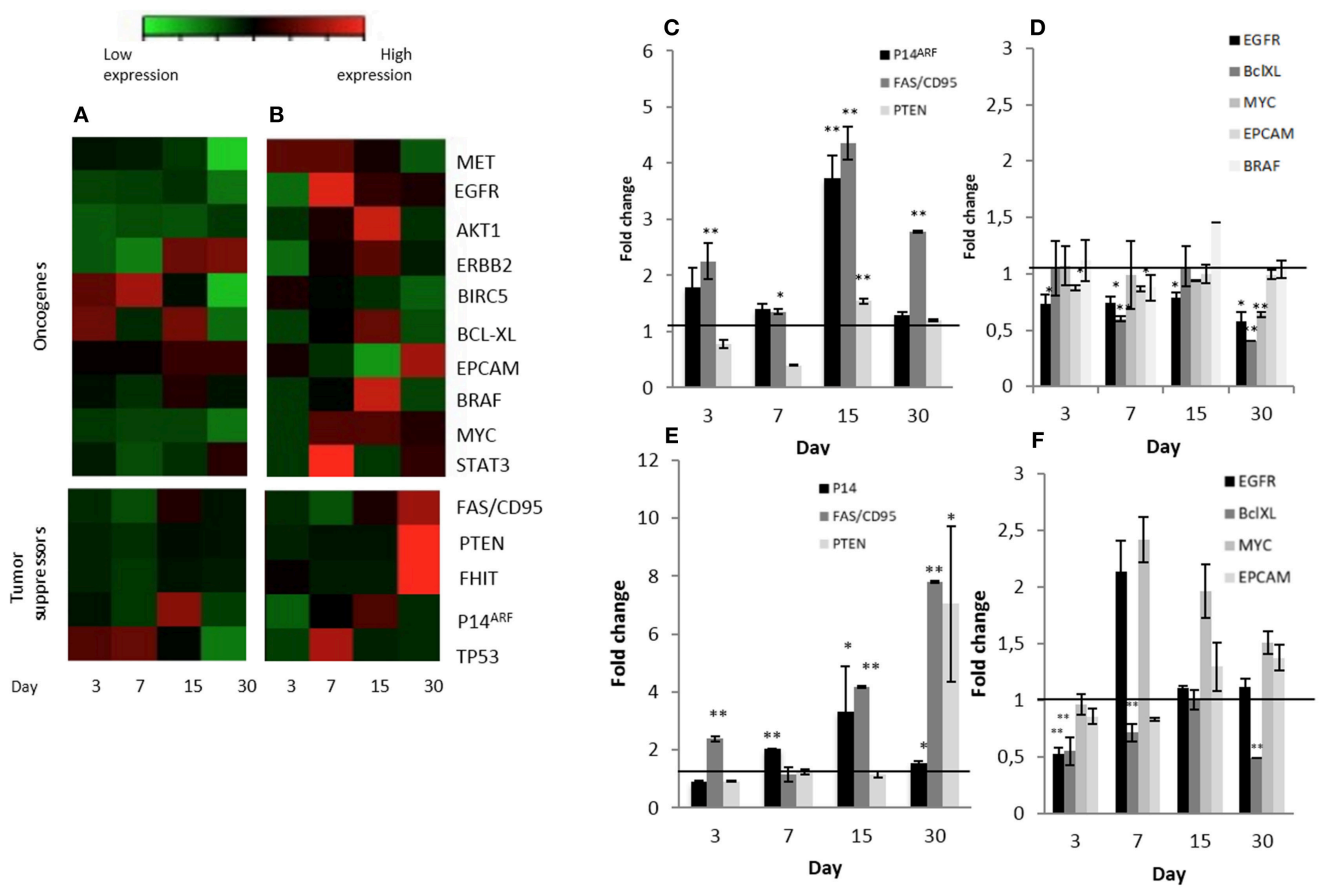
Significant changes tumor suppressors and oncogene expression patterns in Calu-3 cells seeded on micro-scaffolds. Gene expression of all genes tested in **(A)** Calu-3 seeded on micro-scaffolds in comparison with Calu-3 seeded on culture dishes and **(B)** Calu-3 co-cultured with fibroblasts seeded on micro-scaffolds in comparison with Calu-3 co-cultured with fibroblasts seeded on culture dishes. Selected **(C)** tumor suppressors and **(D)** oncogenes with significant differences in Calu-3 seeded on micro-scaffolds in comparison with Calu-3 seeded on culture dishes. Selected **(E)** tumor suppressors and **(F)** oncogenes with significant differences in Calu-3 co-cultured with fibroblasts seeded on micro-scaffolds in comparison with Calu-3 co-cultured with fibroblasts seeded on culture dishes. The line distinguishes between up- and down- regulated genes when compared to cells grown on culture dishes at four time points. Data represent mean ± SD. *p ≤ 0.05, **p ≤ 0.01 (*t*-test).

Three known tumor suppressor genes, FAS/CD95, P14^ARF^ and PTEN, increased significantly their expression in Calu-3 cells seeded on lung-derived micro-scaffolds in comparison with its cell control grown in culture dishes (Figure 4C). FAS/CD95, is an important suppressor as apoptosis inductor and often downregulated in lung adenocarcinoma cells (30). P14ARF is important for the activation of the well-known tumor suppressor P53 (31) and PTEN is a key maintainer of genomic stability and one of the main antagonists of the phosphatidylinositol 3-kinase (PI3K)-AKT serine/threonine kinase 1 oncogenic pathway (32).

Moreover, a significant down-regulation of some evaluated oncogenes, EGFR, BCL-XL, MYC and EPCAM, was observed when Calu-3 seeded on the micro-scaffolds were compared with Calu-3 cells seeded on culture dishes (Figure 4D). EGFR, can affect cell proliferation, invasion and angiogenesis (33). BCL-XL promotes cell survival by indirect inhibition of the activity of a pro-apoptotic pathway and is related with proliferation, survival and differentiation of lung cancer cells (34). MYC has been associated to abnormal autonomous proliferation and growth, angiogenesis and suppression of host immune responses (35) and overexpression of EPCAM has been associated epithelial tumors, specifically with an increase in migratory potential (36).

### Effect of Adding Stromal Fibroblasts to Calu-3 Seeded on Natural Micro-Scaffolds

In an attempt to represent the *in vivo* situation even closer, stromal cells were also seeded to the micro-scaffolds previously seeded with Calu-3 cells. The constructs were cultured for different time periods and expression of all genes mentioned before, were tested again but this time the comparison was with Calu-3 co-cultured with primary fibroblasts seeded on standard plastic culture dishes. As in the group of micro-scaffolds seeded only with Calu-3 cells, micro-scaffolds with Calu-3 plus primary fibroblasts, the same tumor suppressors, *FAS/CD95, P14*^*ARF*^ and *PTEN* were significantly up-regulated in compared with Calu-3 co-cultured with fibroblast seeded on plastic culture dishes as shown in Figures 4B,E. In the presence of fibroblasts, only two genes showed a significant down-regulation, EGFR at day 3 and BCL-XL during 1 month. However, other oncogenes, MYC and EPCAM, were up-regulated (Figures 4B,F).

## Discussion

Epithelial cells are always supported by a connective tissue or stroma. Thus, epithelial-stromal interactions must be necessary for homeostasis in any epithelial-containing organ. Here we have tried to reconstruct -as far as possible- the microenvironment naturally encountered by lung alveolar cells. Previously it has been demonstrated that our lung-derived micro-scaffolds not only supported cell growth of primary and even of expanded alveolar lung cells but also caused the cells to re-differentiate and to form organized alveolar structures (26). We have taken advantage of this system and seeded instead of normal alveolar cells, transformed lung-derived Calu-3 cells, in order to study the influence of normal stroma on the morphology and function of tumorigenic cells. We believe that this is a great model in which carcinogenic cells are examined *in vitro* in an “*in-vivo*” like near normal context.

As a proof of concept, we demonstrated that Calu-3 cells can populate natural lung-derived micro-scaffolds and to organize themselves into a continuous one-layer epithelium lining the alveolar cavities (Figures 2, 3). The apical surface of the cells was found to be covered by microvilli while the basal surface of the cells was found to be lined by a basement membrane (marked by arrows on Figure 3C). These results show acellular micro-scaffolds of appropriate dimensions and that highly preserve the structure of the original stroma can significantly alter the transformed phenotype of even the transformed cell line -Calu-3-. The fact that such a degree of interaction and organization was achieved between the epithelial cells and the stroma *in vitro* should allow to study carcinomas *in vitro* in a near *in vivo* like conditions.

In conceptual agreement, we also show that the morphological changes observed in the organization of the cells within the micro-scaffolds *in vitro* was also accompanied by changes in transcription levels of key genes studied as examples. Our results revealed up-regulation mainly of three tumor suppressor genes (Figures 4A,C) and down regulation of several oncogenes (Figures 4A,D) by Calu-3 cells when they were seeded on lung-derived micro-scaffolds as compared to standard calu-3 cells grown on plastic. This could suggest that the natural lung-derived scaffolds with a conserved structural and protein composition have some influence over the malignant phenotype displayed by Calu-3 cells aberrant signaling.

The acellular micro-scaffolds used here have been characterized previously and found to preserve the key structural features and protein components of the ECM from their original organs (29). Yet, they have been found to be completely devoid of cells and to contain <1per cent of residual DNA (29). In spite of this it should be pointed out that the model proposed allows to introduce stromal cells such as fibroblasts and even endothelial cells if the study requires it (as indicated in Figure 1). In this report, addition of fibroblasts as well as Calu-3 cells to the micro-scaffolds lead to and even higher up-regulation of the key tumor suppressors genes studied (Figures 4B,E). However, it should be noted, as shown in Figures 4B,F, the gene expression profile of some oncogenes increased rather that decreased when fibroblasts were also introduced into the construct. Since PCR primers only amplify human derived cDNA, clearly the changes observed on gene expression profiles can only be attributed to the Calu-3 cells themselves.

The study of epithelium-stroma communication and specifically the role of microenvironment in carcinogenesis has gotten further recognition due to their importance in therapeutic issues (37). However, to try to mimic the complete tumor context and all the components involved in this complex interaction has been a challenge for researchers in this field and there is a considerable effort to recreate the ECM structure and its protein composition (38). Currently as 3D tumor model to study cancer *in vitro* organoids and spheroids are being widely used and have demonstrated cell proliferation, organization and differentiation with the addition of growth factors (38). Nevertheless, we consider that these models do not mimic the conditions found in nature.

Additionally, other authors have proposed acellular organs recellularized with both normal (39) and carcinogenic cell lines (25) to engineer a lung *in vitro*. In these kind of models for a proper growth of the cells, it is necessary a bioreactor which provides sufficient oxygen and nutrient by diffusion into the lung (25, 39). Using bioreactors to repopulate a complete rat lung, authors have used between 10 × 10^6^ and 50 × 10^6^ cells (25, 39). Specifically when human A549, H460, or H1299 lung cancer cells lines were seeded in a complete decellularized rat lung, they developed tumor nodules (25). We consider this form of seeding does not allow having a control about how many cells are finally into the decellularized lung and this could overpopulate the matrix and cause said tumor nodules.

In comparison with acellular organ models mentioned above, the model proposed here, also contains the structure of the extracellular matrix and preserves the highly complex combination of proteins and growth factors (26–28) present in the organ from which it is derived. However, due to its dimensional properties−300 microns in thickness- our scaffolds guarantee that any seeded cell can obtain gases and nutrients by simple diffusion and thus eliminating the need for vascularization or a bioreactor. Cells are seeded directly on the scaffolds and its proportions allow seeding in a controlled way the right amount of cells. Its characteristics make it a practical model for an adequate cells cultivation and maintenance.

In contrast to the cancer cell lines seeded on whole decellularized organ models, when Calu-3 cells were seeded in our scaffolds alone or in co-culture with normal fibroblasts, they not only were well organized in typical lung structures but also they did not form any tumor nodules and showed a less aberrant phenotype in comparison with their control seeded on culture dishes. However, deeper molecular analysis should be conducted to gain a better understanding about carcinoma cells and ECM interactions.

Clearly, a strong limitation of the work presented here, is that a highly “abnormal” cell line was used as our case study. However, the results showed here introduce for the first time a system where carcinogenic cells can be tested in a closely natural but normal environment. Surprisingly our results not only display a proof of principle for the concepts outlined but, not unexpectedly, show that such a “benevolent micro-environment” can even affect the malignant phenotype of a transformed cell line.

The complex modular model presented here allows to specifically dissect the effect of many of its key components. For instance it not only allows studying the behavior of carcinogenic cells in a native normal stroma but also the interaction between normal epithelial cells and a carcinogenic stroma which may or may not contain stromal, endothelial and/or immunomodulatory cells.

## Contribution to the Field Statement

We present here a system in which the various constituting epithelial and/or stromal structural and cellular components of an *in vivo* carcinoma are incorporated. The model takes into consideration the fact that in nature, epithelial cells are always supported by a connective tissue or stroma. The model builds on micro-scaffolds of microscopic dimensions and allows for different types of cells to be seeded on them in order to recreates as far as possible the *in vivo* conditions. An important feature of the micro-scaffolds which constitute the basis for our model system is that they are never more than 300 microns in thickness. This guarantees that any seeded cell can obtain gases and nutrients by simple diffusion and thus eliminating the need for vascularization or a bioreactor. We consider that the main advantage over current *in vitro* tumor models is that our tumor micro-model will allow to create a near normal whole tumor microenvironment where interactions between epithelial cells and their all stromal components during carcinogenesis can be studied.

## Ethics Statement

Animal experiments were performed under the guidelines and approval of the Animal Care and Use Committee, The Faculty of Science of the Hebrew University, Jerusalem, Israel (Permit number NS-17-15219-3).

## Author Contributions

SC and EM: conception and design of study. SC, YS, and AA: acquisition of data. SC, YS, AA, and EM: analysis and/or interpretation of data. SC and EM: drafting the manuscript, and revising the manuscript critically for important intellectual content.

### Conflict of Interest Statement

The authors declare that the research was conducted in the absence of any commercial or financial relationships that could be construed as a potential conflict of interest.
